# Relationship of *Helicobacter pylori* Infection with Nonalcoholic Fatty Liver Disease: A Meta-Analysis

**DOI:** 10.1155/2023/5521239

**Published:** 2023-01-25

**Authors:** Guangqin Xu, Shaoze Ma, Liyan Dong, Nahum Mendez-Sanchez, Hongyu Li, Xingshun Qi

**Affiliations:** ^1^Department of Gastroenterology, General Hospital of Northern Theater Command, Shenyang 110840, China; ^2^Postgraduate College, Dalian Medical University, Dalian 116044, China; ^3^Postgraduate College, China Medical University, Shenyang 110122, China; ^4^Liver Research Unit, Medica Sur Clinic & Foundation, Faculty of Medicine, National Autonomous University of Mexico, Mexico City, Mexico

## Abstract

**Background and Aims:**

*Helicobacter pylori* (*H. pylori*) and nonalcoholic fatty liver disease (NAFLD) have become increasingly recognized, both of which affect human health globally. The association of *H. pylori* infection with NAFLD remains unclear.

**Methods:**

PubMed, EMBASE, and Cochrane Library databases were searched. Only a random-effects model was used. Odds ratios (ORs) and risk ratios (RRs) with 95% confidence intervals (CIs) were calculated for the combined estimates of raw data. Adjusted ORs (aORs) and hazard ratios (aHRs) with 95% CIs were calculated for the combined estimates of data adjusted for confounders.

**Results:**

Thirty-four studies with 218573 participants were included. Based on unadjusted data from 26 cross-sectional studies and 3 case-control studies, *H. pylori* infection was significantly associated with the presence of NAFLD (OR = 1.26, 95% CI = 1.17–1.36, *P* < 0.001). Based on adjusted data from 15 cross-sectional studies and 1 case-control study, *H. pylori* infection was significantly associated with the presence of NAFLD (aOR = 1.25, 95% CI = 1.08–1.44, *P* < 0.001). Compared with control subjects without NAFLD, patients with moderate (OR = 1.67, 95% CI = 1.17–2.39, *P* = 0.005) and severe (OR = 1.71, 95% CI = 1.30–2.24, *P* < 0.001) NAFLD, but not those with mild NAFLD (OR = 1.14, 95% CI = 0.9–1.45, *P* = 0.286), had significantly higher proportions of *H. pylori* infection. The association of *H. pylori* infection with the occurrence of NAFLD was statistically significant based on adjusted data from 3 cohort studies (aHR = 1.18, 95% CI = 1.05–1.34, *P* = 0.007), but not based on unadjusted data from 3 cohort studies (RR = 1.41, 95% CI = 0.80–2.48, *P* = 0.237).

**Conclusion:**

*H. pylori* infection is associated with NAFLD, especially moderate and severe NAFLD. The impact of *H. pylori* eradication on the prevention of NAFLD should be further explored.

## 1. Introduction

*Helicobacter pylori* (*H. pylori*) infects about half of the world's population, especially people living in developing countries and poor socioeconomic countries [1–4]. Considering such a high infection rate, it is recognized as a major public health problem worldwide [2]. *H. pylori* is a main pathogenic factor for chronic gastritis, peptic ulcers, gastric cancer, and gastric mucosa-associated lymphoid tissue (MALT) lymphoma [5]. *H. pylori* infection may also disturb a series of biological processes and determine or influence the development and severity of various extragastric diseases [6], such as insulin resistance, metabolic syndrome, diabetes, nonalcoholic fatty liver disease (NAFLD), vitamin B_12_ deficiency, cardiovascular, neurological, dermatological, and ophthalmic diseases [7].

NAFLD is considered a major hepatic manifestation of metabolic syndrome [8] and includes a full spectrum of fatty liver disease from simple hepatic steatosis or nonalcoholic fatty liver (NAFL) to nonalcoholic steatohepatitis (NASH) and cirrhosis [9]. NAFLD has become the most common type of chronic liver disease, with a global prevalence of approximately 25% [10]. Recently, a new nomenclature of metabolic dysfunction-associated fatty liver disease (MAFLD) has been proposed to replace NAFLD with updated diagnostic criteria and recognized by worldwide experts [11, 12]. Regardless, there is now growing evidence that the development of NAFLD is associated with gut microbiota imbalance [13]. Some studies suggested an association between *H. pylori* infection and NAFLD, and the presence of *H. pylori* or *Helicobacter* species has been observed in liver specimens from patients with various liver diseases [14–16]. However, others indicated no correlation between them [17–19]. Considering the importance of understanding potential risk factors for NAFLD on its management, we have conducted an updated meta-analysis of studies published to date to explore the association between *H. pylori* infection with NAFLD.

## 2. Methods

### 2.1. Registration

This study was registered on the PROSPERO with a registration number CRD42021247307.

### 2.2. Literature Search

The relevant publications were searched via *PubMed*, *Cochrane library*, and *EMBASE* databases. The search terms were as follows: (“HP” or “*H. pylori*” or “*Helicobacter pylori*” or “Helicobacter infection” or “Helicobacter”) and (“Nonalcoholic fatty liver disease” or “Fatty liver” or “Nonalcoholic fatty liver” or “Nonalcoholic steatohepatitis” or “NAFLD” or “NASH” or “NAFL”). There was no language restriction. The last search was conducted on July 14, 2022.

### 2.3. Eligibility Criteria

Inclusion criteria were as follows: (1) eligible studies should include patients who were diagnosed with NAFLD and detected the *H. pylori* infection, (2) eligible studies should clearly report the diagnostic methods of *H. pylori* infection and NAFLD, (3) eligible studies should provide the number of patients with positive and negative *H. pylori* infection in NAFLD patients and control subjects without NAFLD or report the odds ratios (ORs) or hazard ratios (HRs) with 95% confidence intervals (CIs) to evaluate the association between *H. pylori* infection and NAFLD, and (4) age >18 years old. If multiple publications were available for the same study, only the publication with the most complete data would be included.

Exclusion criteria were as follows: (1) duplicated studies, (2) consensus, notes, guidelines, editorials, or letters, (3) meta-analyses, reviews, or case reports, and (4) experimental or animal studies.

### 2.4. Data Extraction

The following data were extracted from each study: first author, publication year, study country, study design, publication form (abstract or full text), number of positive and negative *H. pylori* infection in the NAFLD patients and control subjects without NAFLD, and diagnostic methods of *H. pylori* infection and NAFLD. Adjusted ORs (aORs) and adjusted HRs (aHRs) with 95% CIs with confounders adjusted were extracted from the studies where multivariate regression analyses were performed to evaluate the association of *H. pylori* infection with NAFLD. If studies had multiple adjustment models, only the models that reflected the greatest degree of adjustment for confounders and its corresponding aOR and aHR would be further considered in our meta-analysis.

### 2.5. Diagnosis

*H. pylori* infection can be diagnosed by invasive (i.e., endoscopic biopsy) and noninvasive tests (i.e., serology, ^13^C or ^14^C urea breath test, and fecal antigen test). NAFLD can be diagnosed by histology, ultrasonography, and/or surrogate markers of NAFLD, which include hepatic steatosis index (HSI), NAFLD-liver fat score (NAFLD-LFS), and/or fatty liver index (FLI).

### 2.6. Study Quality Assessment

The quality of cohort and case-control studies was assessed by the Newcastle-Ottawa Scale (NOS), a widely used tool for assessing the quality of observational/nonrandomized studies. It has three major domains: (1) selection, (2) comparability, and (3) exposure/outcome. The maximum score is 9. A score of 0–3, 4–6, and 7–9 represents low, moderate, and high quality, respectively. The quality of cross-sectional studies was evaluated by the Agency for Healthcare Research and Quality (AHRQ) with an 11-item checklist. An item would be scored “0,” if its answer was “NO” or “UNCLEAR”; and an item would be scored “1,” if its answer was “YES.” The maximum score is 11. A score of 0–3, 4–7, and 8–11 represents low, moderate, and high quality, respectively.

### 2.7. Statistical Analyses

All statistical analyses were performed using the Stata software version 12.0 (Stata Corp, College Station, USA) and Review Manager software version 5.4 (Cochrane collaboration, the Nordic Cochrane Centre, Copenhagen, Denmark). Only a random-effects model was employed. ORs and RRs with 95% CIs were calculated for the combined estimation of raw data, and aORs and aHRs with 95% CIs were calculated for the combined estimates of data adjusted for confounders. The *I*^2^ statistics and Cochran *Q* test were used to evaluate the heterogeneity, and *P* < 0.1 and/or *I*^2^ > 50% were considered to indicate statistically significant heterogeneity. Subgroup and meta-regression analyses were performed to explore the sources of heterogeneity among the studies with and without adjustment for confounders. They were grouped according to the study design, region, study quality, diagnostic methods of *H. pylori* infection and NAFLD, sample size, adjustment for confounders, and publication form. The interaction between subgroups was tested. Leave-one-out sensitivity analyses were assessed by sequentially omitting one study each time. Publication bias was evaluated by Egger test. *P* < 0.1 was considered as a statistically significant publication bias. In addition, the proportion of *H. pylori* infection was compared according to the severity of NAFLD (i.e., mild, moderate, and severe).

## 3. Results

### 3.1. Study Characteristics

**Figure 1:**
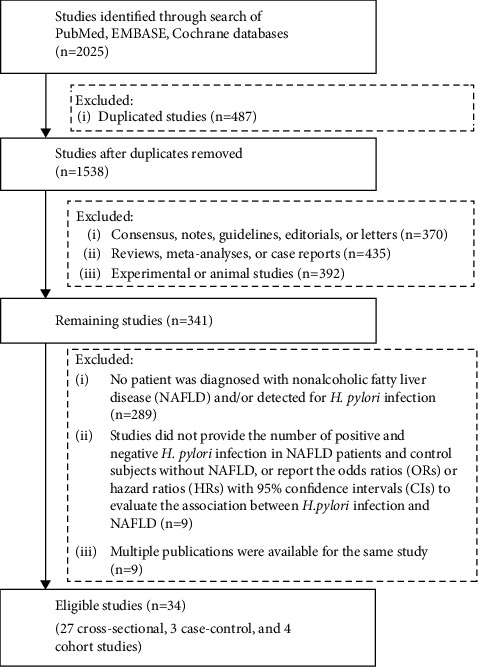
Flowchart of the study selection process.

We initially searched 2025 papers. Finally, 34 studies with 218573 participants were included (Figure 1). Characteristics of included studies are shown in Table 1. Among them, 4 were cohort studies, 3 were case-control studies, and 27 were cross-sectional studies; 3 studies were published as abstracts and 31 as full texts; 25 studies were performed in Asia [17–41], 3 in North America [42–44], 2 in Africa [45, 46], and 4 in Europe [47–50]. The publication date ranged from 2013 to 2022.

### 3.2. Study Quality

Among the cohort and case-control studies, 6 and 1 were of high and moderate quality, respectively (Supplementary Table 1). Among the cross-sectional studies, 16 and 11 were of high and moderate quality, respectively (Supplementary Table 2).

### 3.3. *H. pylori* Infection and Presence of NAFLD

**Figure 2:**
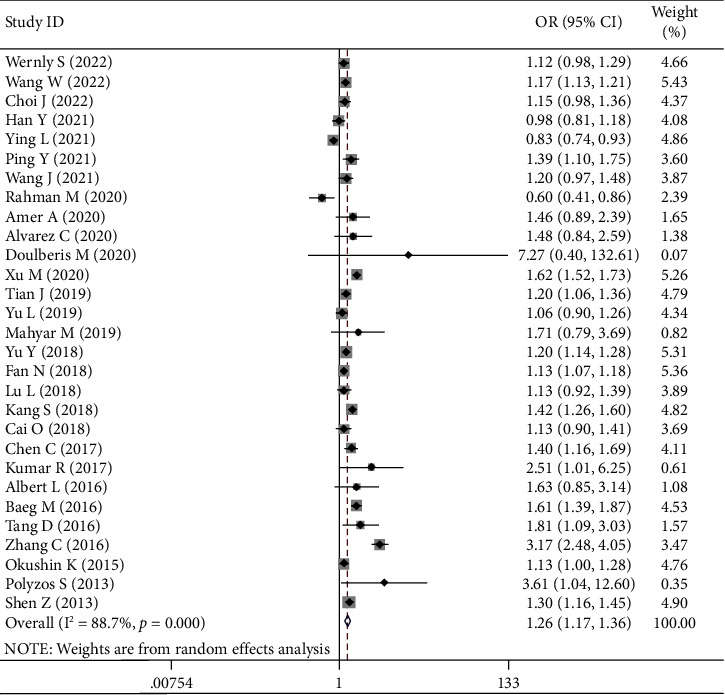
Forest plots for unadjusted data from cross-sectional studies and case-control studies.

Based on the unadjusted data from 26 cross-sectional studies and 3 case-control studies, the meta-analysis showed that *H. pylori* infection was significantly associated with the presence of NAFLD (OR = 1.26, 95% CI = 1.17–1.36, and *P* < 0.001) (Figure 2). Heterogeneity was statistically significant (*I*^2^ = 88.7% nd *P* < 0.001). Such a statistically significant association between them disappeared in the subgroup analyses of studies using the rapid urease test and fecal antigen test to detect *H. pylori* infection, but remained in others. The interaction between subgroups was statistically significant in the subgroup analyses according to the study design (*P* < 0.001) and diagnostic methods of NAFLD (*P* < 0.001), but not in others. Subgroup analyses did not identify any source of heterogeneity (Table 2). Meta-regression analyses showed that the study design (*P* < 0.001) and diagnostic methods of NAFLD (*P* < 0.001) might be the sources of heterogeneity (Supplementary Table 3). Sensitivity analyses did not identify any source of heterogeneity (Supplementary Table 4). Egger test did not show any significant publication bias (*P*=0.294).

**Figure 3:**
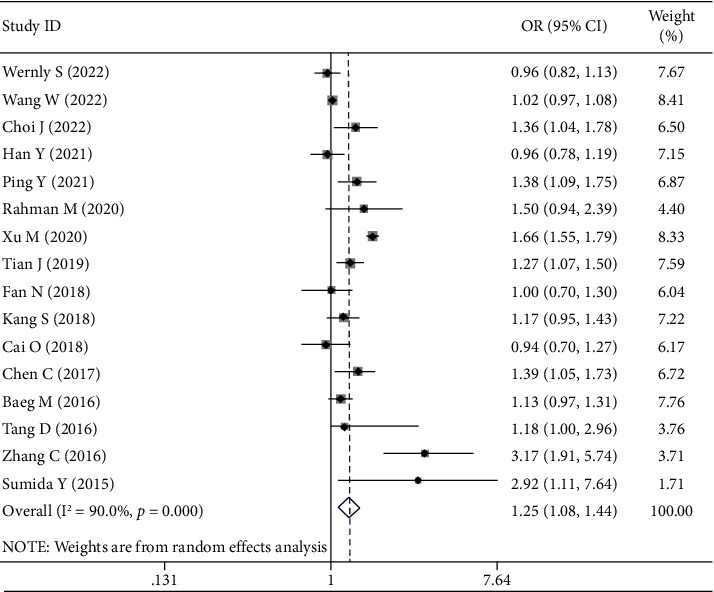
Forest plots for adjusted data from cross-sectional studies and case-control studies.

Based on the adjusted data from 15 cross-sectional studies and 1 case-control study, the meta-analysis showed that *H. pylori* infection was significantly associated with the presence of NAFLD (aOR = 1.25, 95% CI = 1.08–1.44, and *P* < 0.001). Heterogeneity was statistically significant (*I*^2^ = 90% and *P* < 0.001) (Figure 3). Such a statistically significant association between them disappeared in the subgroup analyses of non-Asian studies, those using the rapid urease test to detect *H. pylori* infection, those using surrogate markers for diagnosis of NAFLD, those with a sample size of >5000, and those published as abstracts but remained in others. The interaction between subgroups was statistically significant in the subgroup analyses according to the study design (*P* < 0.001), study quality (*P* < 0.001), diagnostic methods of *H. pylori* (*P*=0.01), and diagnostic methods of NAFLD (*P* < 0.001), but not in others. Subgroup analyses did not identify any source of heterogeneity (Table 3). Meta-regression analyses showed that the study design (*P*=0.015) and diagnostic methods of NAFLD (*P*=0.023) might be the sources of heterogeneity (Supplementary Table 5). Sensitivity analyses did not identify any source of heterogeneity (Supplementary Table 6). Egger test did not show any significant publication bias (*P*=0.591).

### 3.4. *H. pylori* Infection and Severity of NAFLD

The association between *H. pylori* infection and severity of NAFLD was explored in 4 studies (Table 4).

The meta-analysis showed no statistically significant difference in the proportion of *H. pylori* infection between patients with mild NAFLD and those without NAFLD (OR = 1.14, 95% CI = 0.9–1.45, and *P*=0.286). Heterogeneity was statistically significant (*I*^2^ = 95.1% and *P* < 0.001) (Supplementary Figure 1). Because only a small number of studies was included, subgroup analyses were not performed to explore the sources of heterogeneity.

The meta-analysis showed that the proportion of *H. pylori* infection was significantly higher in patients with moderate NAFLD than those without NAFLD (OR = 1.67, 95% CI = 1.17–2.39, and *P*=0.005). Heterogeneity was statistically significant (*I*^2^ = 92.7% and *P* < 0.001) (Supplementary Figure 2). Because only a small number of studies was included, subgroup analyses were not performed to explore the sources of heterogeneity.

The meta-analysis showed that the proportion of *H. pylori* infection was significantly higher in patients with severe NAFLD than those without NAFLD (OR = 1.71, 95% CI = 1.30–2.24, and *P* < 0.001). Heterogeneity was not statistically significant (*I*^2^ = 43.7% and *P*=0.149) (Supplementary Figure 3).

### 3.5. *H. pylori* Infection and Occurrence of NAFLD

Based on the unadjusted data from 3 cohort studies, the meta-analysis showed that *H. pylori* infection was not significantly associated with the occurrence of NAFLD (RR = 1.41, 95% CI = 0.80–2.48, and *P*=0.237). Heterogeneity was statistically significant (*I*^2^ = 98.3% and *P* < 0.001) (Supplementary Figure 4). Because only a small number of studies was included, subgroup analyses were not performed to explore the sources of heterogeneity.

Based on the adjusted data from 3 cohort studies, the meta-analysis showed that *H. pylori* infection was associated with the occurrence of NAFLD (aHR = 1.18, 95% CI = 1.05–1.34, and *P*=0.007). Heterogeneity was statistically significant (*I*^2^ = 70.8% and *P*=0.032) (Supplementary Figure 5). Because only a small number of studies was included, subgroup analyses were not performed to explore the sources of heterogeneity.

## 4. Discussion

Based on the data from cross-sectional studies and case-control studies, *H. pylori* infection was associated with the presence of NAFLD, especially moderate and severe NAFLD. Based on the data from cohort studies, *H. pylori* infection increased the risk of NAFLD occurrence after adjustment for confounders. Notably, seven previous meta-analyses [51–57] also concluded a significant association between *H. pylori* infection with NAFLD. Our current meta-analysis has several advantages compared to previous ones [51–57]. First, there was a more comprehensive collection of eligible studies by expanding the search strategy and updating the final search date. Thus, the number of studies included was larger in the current meta-analysis than in the previous ones. Second, some of the previous meta-analyses did not strictly follow the prespecified inclusion and exclusion criteria to collect all relevant studies. For example, in both meta-analyses by Zhou et al. [51] and Heydari et al. [56], a cross-sectional study by Sumida et al. [40] would have been included based on their eligibility criteria, but neither of them included this study. Third, some of the previous meta-analyses only calculated ORs to evaluate their association. By comparison, the current meta-analysis further pooled HRs to evaluate their cause-effect association. Fourth, the interaction between subgroups was tested to infer whether the impact of *H. pylori* infection on NAFLD was significantly influenced by some confounding factors, which have not been performed in previous meta-analyses yet. Last, the association of *H. pylori* infection with the severity of NAFLD was evaluated in the current meta-analysis, which has not been performed in previous meta-analyses yet.

Metabolic syndrome, including overweight/obesity, type 2 diabetes mellitus (T2DM), and metabolic disorders, is an important pathogenic factor of NAFLD/MAFLD [58, 59]. It is also closely associated with *H. pylori* infection [60]. Notably, insulin resistance (IR) is a key factor in the development of metabolic syndrome [61]. Thus, the pathophysiological interrelationship between *H. pylori* infection and MAFLD/NAFLD may be explained by IR [62–64] (Supplementary Figure 6). First, *H. pylori* infection can stimulate the release of proinflammatory cytokines, such as tumor necrosis factor-*α* (TNF-*α*) [65–67]. TNF-*α* induces serine/threonine-mediated phosphorylation of insulin receptor substrate 1 (IRS-1), which attenuates IRS-1-mediated insulin signaling, leading to the occurrence of IR [68]. Second, *H. pylori* infection causes a decrease in adiponectin levels [48, 69]. Adiponectin can reduce gluconeogenesis and lipogenesis in the liver, and therefore has the effect of an insulin sensitizer to inhibit intrahepatic lipid accumulation [70]. Thus, decreased adiponectin levels would result in increased intrahepatic fat content and IR [71–73]. Third, there is an interaction of reciprocal inhibition between adiponectin and TNF-*α* in terms of their production and action, thereby enhancing IR [74]. Fourth, *H. pylori* infection leads to elevated fetuin-A levels [75]. Fetuin-A can stimulate adipocytes and macrophages to produce proinflammatory cytokines and then induce IR [76, 77].

Besides, their association may be attributed to altered gut microbiota [13]. Chronic *H. pylori* infection causes significant changes in the gut microbiota composition [78]. Gut microbiota can release endotoxin composed of the outer wall of Gram-negative bacteria, which can introduce into the liver directly through the portal vein. Endotoxin can stimulate inflammatory response via Toll-like receptor 4 (TLR4), thereby exacerbating hepatic inflammation [79]. Indeed, some studies have shown that lipopolysaccharide, a surrogate marker of endotoxin, is elevated in patients with NAFLD [80, 81].

Our previous study found a higher rate of *H. pylori* infection in young military personnel than in civilians [82]. This phenomenon is probably explained by the fact that increased mental stress caused by high-intensity military training suppresses the body's humoral and cellular immunity, thereby increasing the risk of *H. pylori* infection. On the other hand, high occupational and personal stress are independent predictors of NAFLD development [83]. Therefore, the association of *H. pylori* infection with the presence of NAFLD may be because both of them have a concomitant predisposing factor (i.e., stress).

Another previous meta-analysis by our group also showed a significant association between *H. pylori* infection and irritable bowel syndrome (IBS) [84]. It should be noted that multiple etiological factors, including obesity, gut microbiota, dietary factors, and immune-mediated causes [85], overlap between IBS and NAFLD. Thus, such factors should not be neglected to explain our current findings about the association of *H. pylori* infection with NAFLD.

Current consensus recommends that dietary modification, exercise, and weight loss as the major treatment option for NAFLD to reduce liver fat and improve IR [86, 87]. Besides, considering that silymarin has antioxidant, anti-inflammatory, immunomodulatory, antifibrotic, and hepatoprotective activities and stimulates protein synthesis and liver tissue regeneration [88], silymarin may be used for the treatment of NAFLD. Notably, it seems that silymarin can also inhibit *H. pylori* activity [89–92]. Therefore, it may be hypothesized that silymarin can be helpful for the treatment of NAFLD and *H. pylori* infection.

Considering an association of *H. pylori* infection with NAFLD, it appears that *H. pylori* eradication is beneficial in preventing NAFLD. However, this is still controversial. A randomized controlled study by Maharshi et al. showed that successful eradication of *H. pylori* in patients with NAFLD resulted in significant improvement in IR [93]. However, a study by Jamali et al. found that *H. pylori* eradication may not influence liver fat content, liver function tests, lipid profile, IR, and anthropometric measurements in patients with dyspeptic NAFLD [94]. Therefore, whether *H. pylori* eradication influences the occurrence or progression of NAFLD needs to be confirmed by more studies in the future.

Consensus and practice guidelines recommend bismuth quadruple therapy, which consists of a proton pump inhibitor, a bismuth, and two antibiotics, for a duration of 10–14 days as the first-line treatment of *H. pylori*. Commonly used antibiotics include amoxicillin, clarithromycin, and metronidazole [95–98]. However, considering an increased burden of multidrug resistant Gram-negative infections [99], some studies have suggested that a combination of tetracycline and tinidazole should achieve a higher rate of *H. pylori* eradication [100–102].

Our meta-analysis has some limitations. First, a majority of these included studies provided only cross-sectional data, which can only establish a possible association between *H. pylori* infection and NAFLD, but not any cause-effect association. Second, only some of these included studies adjusted the confounders in multivariate regression analyses, and the confounders adjusted were inconsistent among them. Third, only a minority of these included studies evaluated the prevalence of *H. pylori* infection according to the severity of NAFLD. Finally, none of these included studies have evaluated the association of *H. pylori* infection with MAFLD.

## 5. Conclusion

There seems to be an association between *H. pylori* infection and NAFLD, but this association was weak. More prospective cohort studies are needed in the future to demonstrate the impact of *H. pylori* infection and its eradication on MAFLD, and experimental studies should also be necessary to elucidate their potential mechanisms. Undoubtedly, these studies may provide promising approaches for the management of MAFLD.

## Figures and Tables

**Table 1:** Characteristics of studies regarding the association of *H. pylori* infection with NAFLD.

First author (year)	Country	Study design	Publication form	Diagnostic methods of *H. pylori*	Diagnostic methods of NAFLD	NAFLD		Control		Adjusted OR/HR (95% CI)	Adjusted confounders
						HP^+^	HP^−^	HP^+^	HP^−^		
Wernly (2022)	Austria	Cross-sectional	Full text	RUT	US	487	1940	532	2379	0.96 (0.82, 1.13)	Age, gender, type 2 diabetes, and LDL
Wang (2022)	China	Cross-sectional	Full text	^13^C-UBT	US	8617	14678	16128	32210	1.02 (0.97, 1.08)	Age, gender, BMI, SBP, DBP, FBG, HbA1C, LDL-C, HDL-C, TG, AST, ALT, GGT, Scr, and BUN
Zhao (2022)	China	Cohort	Full text	^13^C-UBT	US	37	73	169	396	NA	NA
Kim (2022)	South Korea	Cohort	Full text	Serology	US	NA	NA	NA	NA	1.36 (1.18, 1.56)	SBP, FPG, TG, LDL-C, HDL-C, ALT, GGT, and HS-CRP
Choi (2022)	South Korea	Cross-sectional	Full text	Serology	US	660	445	704	548	1.36 (1.04, 1.78)	BMI, HTN, diabetes, dyslipidemia, and smoking
Han (2021)	South Korea	Cross-sectional	Full text	Serology	US	343	528	365	548	0.96 (0.78, 1.19)	Age, gender, HTN, diabetes, BMI, fasting glucose, TG, HDL-C, and LSM
Ying (2021)	China	Cross-sectional	Full text	^13^C-UBT	US	1412	2543	685	1025	NA	NA
Ping (2021)	China	Cross-sectional	Full text	^13^C-UBT	US	230	299	234	422	1.38 (1.09, 1.75)	Age, carotid plaque status, ALT, AST, UA, FPG, TC, TG, SBP, DBP, LDL-C, and BMI
Wang (2021)	China	Cross-sectional	Full text	^13^C-UBT	US	199	306	490	903	NA	NA
Rahman (2020)	Bangladesh	Cross-sectional	Full text	Serology	US	62	79	356	270	1.50 (0.94, 2.39)	Age, gender, religion, BMI, DM, marital status, smoking, occupation, monthly income, MS, and education
Amer (2020)	Egypt	Cross-sectional	Full text	SAT	US	442	82	96	26	NA	NA
Alvarez (2020)	Guatemala	Cross-sectional	Full text	Serology	FLI > 60 and HSI > 36	222	29	145	28	NA	NA
Doulberis (2020)	Switzerland	Case-control	Full text	RUT	Liver biopsy	15	40	0	9	NA	NA
Xu (2020)	China	Cross-sectional	Full text	Serology	US	2516	2309	5287	7859	1.66 (1.55, 1.79)	Age, gender, underlying diseases, and MS
Tian (2019)	China	Cross-sectional	Full text	^13^C-UBT	US	1022	842	1115	1102	1.27 (1.07, 1.50)	Age, gender, education level, smoking, HTN, diabetes, dyslipidemia, BMI, ALT, AST, AKP, TBIL, UA, and urea
Yu (2019)	China	Cross-sectional	Full text	RUT	US	583	851	379	589	NA	NA
Mahyar (2019)	Iran	Cross-sectional	Full text	Serology and SAT	US	22	43	15	50	NA	NA
Abdel-Razik (2018)	Egypt	Cohort	Full text	SAT	US, HSI > 36 and NAFLD-LFS > −0.640	23	0	148	198	1.08 (1.02, 1.25)	Age, gender, BMI, smoking, crowding index, education level, regular exercise, CRP, IL-6, TNF-*α*, HOMA-IR, FPG, TC, HDL-C, LDL-C, TG, and UA
Yu (2018)	China	Cross-sectional	Full text	^14^C-UBT	US	3132	4460	4716	8081	NA	NA
Fan (2018)	China	Cross-sectional	Full text	^14^C-UBT	US	3905	5768	6943	11554	1.00 (0.70, 1.30)	Age, gender, BMI, SBP, DBP, FPG, HbA1c, TG, TC, LDL-C, HDL-C, UA, and Scr
Lu (2018)	China	Cross-sectional	Full text	^13^C-UBT	US	199	397	390	881	NA	NA
Kang (2018)	USA	Cross-sectional	Full text	Serology	US	658	1065	1115	2566	1.17 (0.95, 1.43)	Age, gender, race ethnicity, income, diabetes, HTN, smoking, waist circumference, alcohol and caffeine consumption, TC, HDL-C, and transferrin saturation
Cai (2018)	China	Cross-sectional	Full text	^13^C-UBT	US	145	288	500	1118	0.94 (0.70, 1.27)	Gender, BMI, TG, HDL-C, and FPG
Kim (2017)	South Korea	Cohort	Full text	Serology	US	2080	1301	7838	5809	1.16 (1.05, 1.30)	Age, gender, BMI, year of screening exam, smoking status, alcohol intake, regular exercise, and education level, HS-CRP, HOMA-IR, SBP, FPG, TG, LDL-C, HDL-C, AST, ALT, and GGT
Chen (2017)	China	Cross-sectional	Full text	^13^C-UBT	US	313	290	723	937	1.39 (1.05, 1.73)	Age, gender, UA, AST, ALT, GGT, TG, BMI, waist circumference, and HbA1C
Kumar (2017)	India	Cross-sectional	Abstract	RUT	US	11	16	20	73	NA	NA
Albert (2016)	Spain	Cross-sectional	Full text	RUT	Liver biopsy	264	110	25	17	NA	NA
Baeg (2016)	South Korea	Cross-sectional	Full text	^13^C-UBT	HSI > 36	505	440	1131	1587	1.13 (0.97, 1.31)	Age, gender, smoking, and HS-CRP
Tang (2016)	USA	Cross-sectional	Abstract	RUT, serology or SAT	US or liver biopsy	49	73	40	108	1.18 (1.00, 2.96)	Age, gender, and statin use
Zhang (2016)	China	Case-control	Full text	^14^C-UBT	Liver biopsy	300	300	144	456	3.17 (1.91, 5.74)	Gender and geriatric diseases
Okushin (2015)	Japan	Cross-sectional	Full text	Serology	US	523	1279	926	2561	NA	NA
Sumida (2015)	Japan	Cross-sectional	Full text	Serology	Liver biopsy	NA	NA	NA	NA	2.92 (1.11, 7.64)	Age, gender, BMI, dyslipidemia, HTN, and diabetes
Polyzos (2013)	Greece	Case-control	Full text	Serology	Liver biopsy	23	5	14	11	NA	NA
Shen (2013)	China	Cross-sectional	Abstract	Serology	US	566	1307	1804	5414	NA	NA

AKP: alkaline phosphatase, ALT: alanine aminotransferase, AST: aspartate aminotransferase, BMI: basal metabolic index, BP: blood pressure, BUN: blood urea nitrogen, CRP: C-reactive protein, CI: confidence intervals, DBP: diastolic blood pressure, DM: diabetes mellitus, FBG/FPG: fasting plasma glucose, FLI: fatty liver index, GGT: gamma-glutamyl transpeptidase, HbA1c: glycosylated hemoglobin, HDL-C: high-densitylipoprotein-cholesterol, HP: *Helicobacter pylori*, HS-CRP: high-sensitivityC-reactive protein, HOMA-IR: homeostatic model assessment-insulin resistance, HR: hazard ratio, HSI: hepatic steatosis index, HTN: hypertension, IL-6: interleukin-6, LDL: low-density lipoprotein, LDL-C: low-densitylipoprotein-cholesterol, LSM: liver stiffness measurements, MS: metabolic syndrome, NA: not available, NAFLD: nonalcoholic fatty liver disease, NAFLD-LFS: NAFLD-liver fat score, OR: odds ratio, PG: pepsinogen, RUT: rapid urease test, SAT: stool antigen test, Scr: serum creatinine, SBP: systolic blood pressure, TBIL: total bilirubin, TC: total cholesterol, TG: triglycerides, TNF-*α*: tumor necrosis factor-alpha, UA: uric acid, UBT: urea breath test, US: ultrasonography, and USA: the United States of America.

**Table 2:** Meta-analysis regarding the association of *H. pylori* infection with NAFLD in studies unadjusted for confounders.

Groups	No. studies	OR (95% CI)	Heterogeneity		*P*_interaction_
			*I* ^2^ (%)	*P* value	
Study design					<0.001
Cross-sectional	26	1.21 (1.13–1.30; *P* < 0.001)	86.40	<0.001	
Case-control	3	3.20 (2.52–4.07; *P* < 0.001)	0.00	0.839	
Region					0.17
Asia	21	1.23 (1.14–1.34; *P* < 0.001)	91.30	<0.001	
Non-Asia	8	1.40 (1.18–1.66; *P* < 0.001)	45.70	0.075	
Study quality					0.96
Moderate-quality	11	1.27 (1.06–1.52; *P*=0.01)	77.40	<0.001	
High-quality	18	1.27 (1.17–1.39; *P* < 0.001)	91.20	<0.001	
Diagnostic methods of *H. pylori*					0.75
UBT	12	1.27 (1.15–1.39; *P* < 0.001)	91.00	<0.001	
RUT	5	1.16 (0.98–1.39; *P*=0.088)	35.60	0.184	
Serology	9	1.21 (1.03–1.42; *P*=0.021)	89.00	<0.001	
SAT	1	1.46 (0.89–2.39; *P*=0.133)	0.00	—	
Diagnostic methods of NAFLD					<0.001
US	22	1.18 (1.10–1.27; *P* < 0.001)	87.30	<0.001	
Liver biopsy	4	2.75 (1.87–4.06; *P* < 0.001)	23.70	0.269	
Surrogate markers of NAFLD^*∗*^	2	1.60 (1.39–1.85; *P* < 0.001)	0.00	0.772	
Sample size					0.21
>5000	9	1.20 (1.09–1.32; *P*=0.008)	94.10	<0.001	
<5000	20	1.34 (1.16–1.55; *P* < 0.001)	82.20	<0.001	
Publication form					0.25
Full text	26	1.25 (1.15–1.35; *P* < 0.001)	89.60	<0.001	
Abstract	3	1.51 (1.10–2.09; *P*=0.011)	41.50	0.181	

^*∗*^Surrogate markers of NAFLD include FLI > 60, HSI > 36, or NAFLD-LFS > −0.640. CI: confidence intervals, FLI: fatty liver index, HSI: hepatic steatosis index, NAFLD: nonalcoholic fatty liver disease, NAFLD-LFS: NAFLD-liver fat score, OR: odds ratio, RUT: rapid urease test, SAT: stool antigen test, UBT: urea breath test, and US: ultrasonography.

**Table 3:** Meta-analysis regarding the association of *H. pylori* infection with NAFLD in studies adjusted for confounders.

Groups				No. studies	aOR (95% CI)			Heterogeneity		*P*_interation_
								*I* ^2^ (%)	*P* value	
Study design										<0.001
Cross-sectional				15	1.20 (1.05–1.38; *P*=0.009)			89.8%	<0.001	
Case-control				1	3.17 (1.83–5.50; *P* < 0.001)			—	—	
Region										0.05
Asia				13	1.30 (1.10–1.53; *P*=0.002)			91.5%	<0.001	
Non-Asia				3	1.05 (0.91–1.21; *P*=0.503)			18.00%	0.295	
Study quality										<0.001
Moderate-quality				1	2.21 (1.18–4.12; *P*=0.029)			—	—	
High-quality				15	1.23 (1.07–1.42; *P*=0.004)			90.4%	<0.001	
Diagnostic methods of *H. pylori*										0.01
UBT				8	1.21 (1.05–1.40; *P*=0.009)			78.1%	<0.001	
Serology				6	1.38 (1.06–1.73; *P*=0.015)			84.7%	<0.001	
RUT				1	0.96 (0.82–1.13; *P*=0.618)			—	—	
Diagnostic methods of NAFLD										<0.001
US				12	1.19 (1.02–1.39; *P*=0.028)			91.8%	<0.001	
Liver biopsy				2	3.11 (1.93–5.01; *P* < 0.001)			0.00%	0.885	
Surrogate markers of NAFLD^*∗*^				1	1.13 (0.97–1.31; *P*=0.111)			—	—	
Sample size										0.25
>5000				5	1.15 (0.87–1.50; *P*=0.325)			96.7%	<0.001	
<5000				11	1.29 (1.12–1.48; *P* < 0.001)			63.4%	0.002	
Confounders adjusted										0.05
Full adjusted^*∗*^				10	1.16 (1.04–1.29; *P*=0.006)			61.5%	0.005	
No full adjusted				6	1.46 (1.08–1.98; *P*=0.013)			91.6%	<0.001	
Publication form										0.35
Full text				15	1.25 (1.08–1.45; *P*=0.002)			90.6%	<0.001	
Abstract				1	1.18 (0.69–2.03; *P*=0.550)			—	—	

^*∗*^Surrogate markers of NAFLD include FLI > 60, HSI > 36, or NAFLD-LFS > −0.640. ^*∗*^Full adjusted: at least age, gender, BMI, and/or smoking, as well as three additional risk factors were adjusted. BMI: basal metabolic index, CI: confidence intervals, FLI: fatty liver index, HSI: hepatic steatosis index, NAFLD: nonalcoholic fatty liver disease, NAFLD-LFS: NAFLD-liver fat score, aOR: adjusted odds ratio, RUT: rapid urease test, UBT: urea breath test, and US: ultrasonography.

**Table 4:** *H. pylori* infection and NAFLD severity.

First author (year)	NAFLD			Non-NAFLD
	Mild	Moderate	Severe	
	HP^+^/HP^−^	HP^+^/HP^−^	HP^+^/HP^−^	HP^+^/HP^−^
Wang (2022)	6711/11549	1852/3044	54/85	16128/32210
Wang (2021)	119/187	68/106	12/13	490/903
Amer (2020)	80/49	202/10	160/23	96/26
Xu (2020)	1901/1825	407/323	208/161	5287/7859

HP: *Helicobacter pylori* and NAFLD: nonalcoholic fatty liver disease.

## Data Availability

Data sharing is not applicable to this article as no new data were created in this study.

## Supplementary Materials

Supplementary Materials The paper includes supplementary tables 1–6 as supplementary materials. Their descriptions are as follows: Supplementary Table 1: quality of cohort and case-control studies. Supplementary Table 2: quality of cross-sectional studies. Supplementary Table 3: results of meta-regression analyses regarding the association of *H. pylori* infection with NAFLD in studies unadjusted for confounders. Supplementary Table 4: results of leave-one-out sensitivity analysis in studies unadjusted for confounders. Supplementary Table 5: results of meta-regression analyses regarding the association of *H. pylori* infection with NAFLD in studies adjusted for confounders. Supplementary Table 6: results of leave-one-out sensitivity analysis in studies adjusted for confounders. Supplementary Figure 1: forest plot of the proportion of *H. pylori* infection in patients with mild NAFLD. Supplementary Figure 2: forest plot of the proportion of *H. pylori* infection in patients with moderate NAFLD. Supplementary Figure 3: forest plot of the proportion of *H. pylori* infection in patients with severe NAFLD. Supplementary Figure 4: forest plots for unadjusted data from cohort studies. Supplementary Figure 5: forest plots for adjusted data from cohort studies. Supplementary Figure 6: *H. pylori* infection and the pathophysiological of MAFLD/NAFLD.
