# Supplementary material for: Relationship of Helicobacter pylori Infection with Nonalcoholic Fatty Liver Disease: A Meta-Analysis
Source: Can J Gastroenterol Hepatol. 2023 Jan 25;2023:5521239. doi: 10.1155/2023/5521239 (PMC9891807; doi:10.1155/2023/5521239)
Supplement: Supplementary Materials — The paper includes supplementary tables 1–6 as supplementary materials. Their descriptions are as follows: Supplementary Table 1: quality of cohort and case-control studies. Supplementary Table 2: quality of cross-sectional studies. Supplementary Table 3: results of meta-regression analyses regarding the association of H. pylori infection with NAFLD in studies unadjusted for confounders. Supplementary Table 4: results of leave-one-out sensitivity analysis in studies unadjusted for confounders. Supplementary Table 5: results of meta-regression analyses regarding the association of H. pylori infection with NAFLD in studies adjusted for confounders. Supplementary Table 6: results of leave-one-out sensitivity analysis in studies adjusted for confounders. Supplementary Figure 1: forest plot of the proportion of H. pylori infection in patients with mild NAFLD. Supplementary Figure 2: forest plot of the proportion of H. pylori infection in patients with moderate NAFLD. Supplementary Figure 3: forest plot of the proportion of H. pylori infection in patients with severe NAFLD. Supplementary Figure 4: forest plots for unadjusted data from cohort studies. Supplementary Figure 5: forest plots for adjusted data from cohort studies. Supplementary Figure 6: H. pylori infection and the pathophysiological of MAFLD/NAFLD. [file 5521239.f1.zip › Supplementary Table 1.docx]

| **Supplementary Table 1. Quality of cohort and case-control studies.** | | | | |
| --- | --- | --- | --- | --- |
| **First author (year)** | **Selection (☆☆☆☆)** | **Comparability (☆☆)** | **Exposure/Outcome (☆☆☆)** | **NOS score** |
| Zhao X (2022) | ☆☆☆☆ | ☆/ | ☆☆☆ | 8 |
| Kim J (2022) | ☆☆☆☆ | ☆/ | ☆☆/ | 7 |
| Doulberis M (2020) | ☆☆☆/ | ☆/ | ☆☆/ | 6 |
| Abdel-Razik A (2018) | ☆☆☆/ | ☆/ | ☆☆☆ | 7 |
| Kim T (2017) | ☆☆☆☆ | ☆/ | ☆☆/ | 7 |
| Zhang C (2016) | ☆☆☆☆ | ☆/ | ☆☆/ | 7 |
| Polyzos S (2013) | ☆☆☆☆ | ☆/ | ☆/☆ | 7 |
| **Abbreviations:** NOS: Newcastle–Ottawa Scale. | | | | |
