# Supplementary material for: Relationship of Helicobacter pylori Infection with Nonalcoholic Fatty Liver Disease: A Meta-Analysis
Source: Can J Gastroenterol Hepatol. 2023 Jan 25;2023:5521239. doi: 10.1155/2023/5521239 (PMC9891807; doi:10.1155/2023/5521239)
Supplement: Supplementary Materials — The paper includes supplementary tables 1–6 as supplementary materials. Their descriptions are as follows: Supplementary Table 1: quality of cohort and case-control studies. Supplementary Table 2: quality of cross-sectional studies. Supplementary Table 3: results of meta-regression analyses regarding the association of H. pylori infection with NAFLD in studies unadjusted for confounders. Supplementary Table 4: results of leave-one-out sensitivity analysis in studies unadjusted for confounders. Supplementary Table 5: results of meta-regression analyses regarding the association of H. pylori infection with NAFLD in studies adjusted for confounders. Supplementary Table 6: results of leave-one-out sensitivity analysis in studies adjusted for confounders. Supplementary Figure 1: forest plot of the proportion of H. pylori infection in patients with mild NAFLD. Supplementary Figure 2: forest plot of the proportion of H. pylori infection in patients with moderate NAFLD. Supplementary Figure 3: forest plot of the proportion of H. pylori infection in patients with severe NAFLD. Supplementary Figure 4: forest plots for unadjusted data from cohort studies. Supplementary Figure 5: forest plots for adjusted data from cohort studies. Supplementary Figure 6: H. pylori infection and the pathophysiological of MAFLD/NAFLD. [file 5521239.f1.zip › Supplementary Table 2.docx]

| **Supplementary Table 2. Quality of cross-sectional studies.** | | | | | | | | | | | | | | |
| --- | --- | --- | --- | --- | --- | --- | --- | --- | --- | --- | --- | --- | --- | --- |
| **First author (year)** | **Q1** | **Q2** | **Q3** | **Q4** | **Q5** | **Q6** | **Q7** | **Q8** | **Q9** | **Q10** | **Q11** | **Quality Score** | |  |
| Wernly S (2022) | 1 | 1 | 1 | 0 | 1 | 1 | 1 | 1 | 0 | 1 | 0 | | 8 | |
| Wang W (2022) | 1 | 1 | 1 | 1 | 1 | 1 | 0 | 1 | 0 | 1 | 0 | | 8 | |
| Choi J (2022) | 1 | 1 | 1 | 1 | 1 | 1 | 1 | 1 | 0 | 1 | 0 | | 9 | |
| Han Y (2021) | 1 | 1 | 1 | 1 | 1 | 1 | 1 | 1 | 0 | 1 | 0 | | 9 | |
| Ying L (2021) | 1 | 1 | 1 | 1 | 1 | 0 | 0 | 1 | 0 | 1 | 0 | | 7 | |
| Ping Y (2021) | 1 | 1 | 1 | 1 | 1 | 1 | 1 | 1 | 0 | 1 | 0 | | 9 | |
| Wang J (2021) | 1 | 1 | 1 | 1 | 1 | 1 | 1 | 1 | 0 | 1 | 0 | | 9 | |
| Rahman M (2020) | 1 | 1 | 1 | 1 | 1 | 1 | 1 | 0 | 0 | 1 | 0 | | 8 | |
| Amer A (2020) | 1 | 1 | 1 | 1 | 0 | 0 | 1 | 1 | 0 | 1 | 0 | | 7 | |
| Alvarez C (2020) | 1 | 1 | 1 | 1 | 0 | 1 | 0 | 1 | 0 | 1 | 0 | | 7 | |
| Xu M (2020) | 1 | 0 | 1 | 1 | 1 | 1 | 1 | 1 | 0 | 1 | 0 | | 8 | |
| Tian J (2019) | 1 | 1 | 1 | 1 | 1 | 1 | 0 | 1 | 0 | 1 | 0 | | 8 | |
| Yu L (2019) | 1 | 1 | 1 | 1 | 1 | 1 | 1 | 1 | 0 | 0 | 0 | | 8 | |
| Mahyar M (2019) | 1 | 1 | 1 | 1 | 0 | 1 | 1 | 0 | 0 | 1 | 0 | | 7 | |
| Yu Y (2018) | 1 | 1 | 1 | 1 | 0 | 1 | 1 | 1 | 0 | 1 | 0 | | 8 | |
| Fan N (2018) | 1 | 1 | 1 | 1 | 0 | 1 | 1 | 1 | 0 | 1 | 0 | | 8 | |
| Lu L (2018) | 1 | 1 | 1 | 1 | 0 | 0 | 0 | 0 | 0 | 1 | 0 | | 5 | |
| Kang S (2018) | 1 | 1 | 1 | 1 | 1 | 1 | 1 | 1 | 0 | 0 | 0 | | 8 | |
| Cai O (2018) | 1 | 1 | 1 | 1 | 1 | 1 | 0 | 1 | 0 | 1 | 0 | | 8 | |
| Chen C (2017) | 1 | 1 | 1 | 1 | 0 | 1 | 1 | 1 | 0 | 1 | 0 | | 8 | |
| Kumar R (2017) | 1 | 0 | 1 | 0 | 1 | 0 | 0 | 0 | 0 | 1 | 0 | | 4 | |
| Albert L (2016) | 1 | 1 | 1 | 1 | 0 | 0 | 1 | 0 | 0 | 1 | 0 | | 6 | |
| Baeg M (2016) | 1 | 1 | 1 | 1 | 0 | 1 | 1 | 1 | 0 | 1 | 0 | | 8 | |
| Tang D (2016) | 1 | 0 | 1 | 1 | 0 | 0 | 0 | 1 | 0 | 1 | 0 | | 5 | |
| Okushin K (2015) | 1 | 1 | 1 | 1 | 0 | 0 | 1 | 0 | 0 | 1 | 0 | | 6 | |
| Sumida Y (2015) | 1 | 1 | 1 | 0 | 1 | 1 | 0 | 1 | 0 | 1 | 0 | | 7 | |
| Shen Z (2013) | 1 | 0 | 0 | 0 | 1 | 0 | 0 | 1 | 0 | 1 | 0 | | 4 | |
| Notes: Q1: Define the source of information (survey, record review); Q2: List inclusion and exclusion criteria for exposed and unexposed subjects (cases and controls) or refer to previous publications; Q3: Indicate time period used for identifying patients; Q4: Indicate whether or not subjects were consecutive if not population-based; Q5: Indicate if evaluators of subjective components of study were masked to other aspects of the status of the participants; Q6: Describe any assessments undertaken for quality assurance purposes (e.g., test/retest of primary outcome measurements); Q7: Explain any patient exclusions from analysis; Q8: Describe how confounding was assessed and/or controlled; Q9: If applicable, explain how missing data were handled in the analysis; Q10: Summarize patient response rates and completeness of data collection; Q11: Clarify what follow-up, if any, was expected and the percentage of patients for which incomplete data or follow-up was obtained. | | | | | | | | | | | | | | |
