# Supplementary material for: Relationship of Helicobacter pylori Infection with Nonalcoholic Fatty Liver Disease: A Meta-Analysis
Source: Can J Gastroenterol Hepatol. 2023 Jan 25;2023:5521239. doi: 10.1155/2023/5521239 (PMC9891807; doi:10.1155/2023/5521239)
Supplement: Supplementary Materials — The paper includes supplementary tables 1–6 as supplementary materials. Their descriptions are as follows: Supplementary Table 1: quality of cohort and case-control studies. Supplementary Table 2: quality of cross-sectional studies. Supplementary Table 3: results of meta-regression analyses regarding the association of H. pylori infection with NAFLD in studies unadjusted for confounders. Supplementary Table 4: results of leave-one-out sensitivity analysis in studies unadjusted for confounders. Supplementary Table 5: results of meta-regression analyses regarding the association of H. pylori infection with NAFLD in studies adjusted for confounders. Supplementary Table 6: results of leave-one-out sensitivity analysis in studies adjusted for confounders. Supplementary Figure 1: forest plot of the proportion of H. pylori infection in patients with mild NAFLD. Supplementary Figure 2: forest plot of the proportion of H. pylori infection in patients with moderate NAFLD. Supplementary Figure 3: forest plot of the proportion of H. pylori infection in patients with severe NAFLD. Supplementary Figure 4: forest plots for unadjusted data from cohort studies. Supplementary Figure 5: forest plots for adjusted data from cohort studies. Supplementary Figure 6: H. pylori infection and the pathophysiological of MAFLD/NAFLD. [file 5521239.f1.zip › Supplementary Table 5.docx]

| **Supplementary Table 5. Results of meta-regression analyses regarding the association of *H. pylori* infection with NAFLD in studies adjusted for confounders.** | |
| --- | --- |
| **Variables** | **P value** |
| Study design (Case-control versus cross-sectional) | **0.015** |
| Region (Asia versus non-Asia) | 0.344 |
| Study quality (Moderate-quality versus high-quality) | 0.150 |
| Diagnostic methods of *H. pylori* (UBT versus serology versus RUT) | 0.482 |
| Diagnostic methods of NAFLD (US versus liver biopsy versus surrogate markers of NAFLD*) | **0.023** |
| Sample size (>5000 versus <5000) | 0.400 |
| Confounders adjustment (Full adjusted* versus no full adjusted) | 0.244 |
| Publication form (Full text versus abstract) | 0.940 |
| * Surrogate markers of NAFLD include FLI>60, HSI > 36, or NAFLD-LFS > -0.640. * Full adjusted: at least age, gender, BMI and/or smoking as well as three additional risk factors were adjusted. **Abbreviations:** BMI, basal metabolic index; FLI, fatty liver index; HSI, hepatic steatosis index; NAFLD, nonalcoholic fatty liver disease; NAFLD-LFS, NAFLD-liver fat score; RUT, rapid urease test; UBT, urea breath test; US, ultrasonography. | |
