# Supplementary material for: Relationship of Helicobacter pylori Infection with Nonalcoholic Fatty Liver Disease: A Meta-Analysis
Source: Can J Gastroenterol Hepatol. 2023 Jan 25;2023:5521239. doi: 10.1155/2023/5521239 (PMC9891807; doi:10.1155/2023/5521239)
Supplement: Supplementary Materials — The paper includes supplementary tables 1–6 as supplementary materials. Their descriptions are as follows: Supplementary Table 1: quality of cohort and case-control studies. Supplementary Table 2: quality of cross-sectional studies. Supplementary Table 3: results of meta-regression analyses regarding the association of H. pylori infection with NAFLD in studies unadjusted for confounders. Supplementary Table 4: results of leave-one-out sensitivity analysis in studies unadjusted for confounders. Supplementary Table 5: results of meta-regression analyses regarding the association of H. pylori infection with NAFLD in studies adjusted for confounders. Supplementary Table 6: results of leave-one-out sensitivity analysis in studies adjusted for confounders. Supplementary Figure 1: forest plot of the proportion of H. pylori infection in patients with mild NAFLD. Supplementary Figure 2: forest plot of the proportion of H. pylori infection in patients with moderate NAFLD. Supplementary Figure 3: forest plot of the proportion of H. pylori infection in patients with severe NAFLD. Supplementary Figure 4: forest plots for unadjusted data from cohort studies. Supplementary Figure 5: forest plots for adjusted data from cohort studies. Supplementary Figure 6: H. pylori infection and the pathophysiological of MAFLD/NAFLD. [file 5521239.f1.zip › Supplementary Table 6.docx]

| **Supplementary Table 6. Results of leave-one-out sensitivity analysis in studies adjusted for confounders.** | | | |
| --- | --- | --- | --- |
| **Study omitted** | **aOR (95%CI)** | ***I²* (%)** | **P value** |
| Wernly S (2022) | 1.28 (1.10, 1.48) | 90% | <0.001 |
| Wang W (2022) | 1.27 (1.10, 1.47) | 84% | <0.001 |
| Choi J (2022) | 1.27 (1.07, 1.44) | 91% | <0.001 |
| Han Y (2021) | 1.28 (1.10, 1.48) | 90% | <0.001 |
| Ping Y (2021) | 1.24 (1.07, 1.44) | 91% | <0.001 |
| Rahman M (2020) | 1.24 (1.07, 1.43) | 91% | <0.001 |
| Xu M (2020) | 1.18 (1.07, 1.31) | 69% | 0.001 |
| Tian J (2019) | 1.25 (1.07, 1.45) | 91% | <0.001 |
| Fan N (2018) | 1.27 (1.09, 1.47) | 91% | <0.001 |
| Kang S (2018) | 1.26 (1.08, 1.46) | 91% | <0.001 |
| Cai O (2018) | 1.27 (1.10, 1.47) | 91% | <0.001 |
| Chen C (2017) | 1.24 (1.07, 1.44) | 91% | <0.001 |
| Baeg M (2016) | 1.26 (1.08, 1.47) | 91% | <0.001 |
| Tang D (2016) | 1.25 (1.08, 1.45) | 91% | <0.001 |
| Zhang C (2016) | 1.20 (1.05, 1.38) | 90% | <0.001 |
| Sumida Y (2015) | 1.23 (1.07, 1.42) | 90% | <0.001 |

**Abbreviations:** CI, confidence intervals; aOR, adjusted odds ratio.
