# Supplementary figures and images for: Relationship of Helicobacter pylori Infection with Nonalcoholic Fatty Liver Disease: A Meta-Analysis
Source: Can J Gastroenterol Hepatol. 2023 Jan 25;2023:5521239. doi: 10.1155/2023/5521239 (PMC9891807; doi:10.1155/2023/5521239)

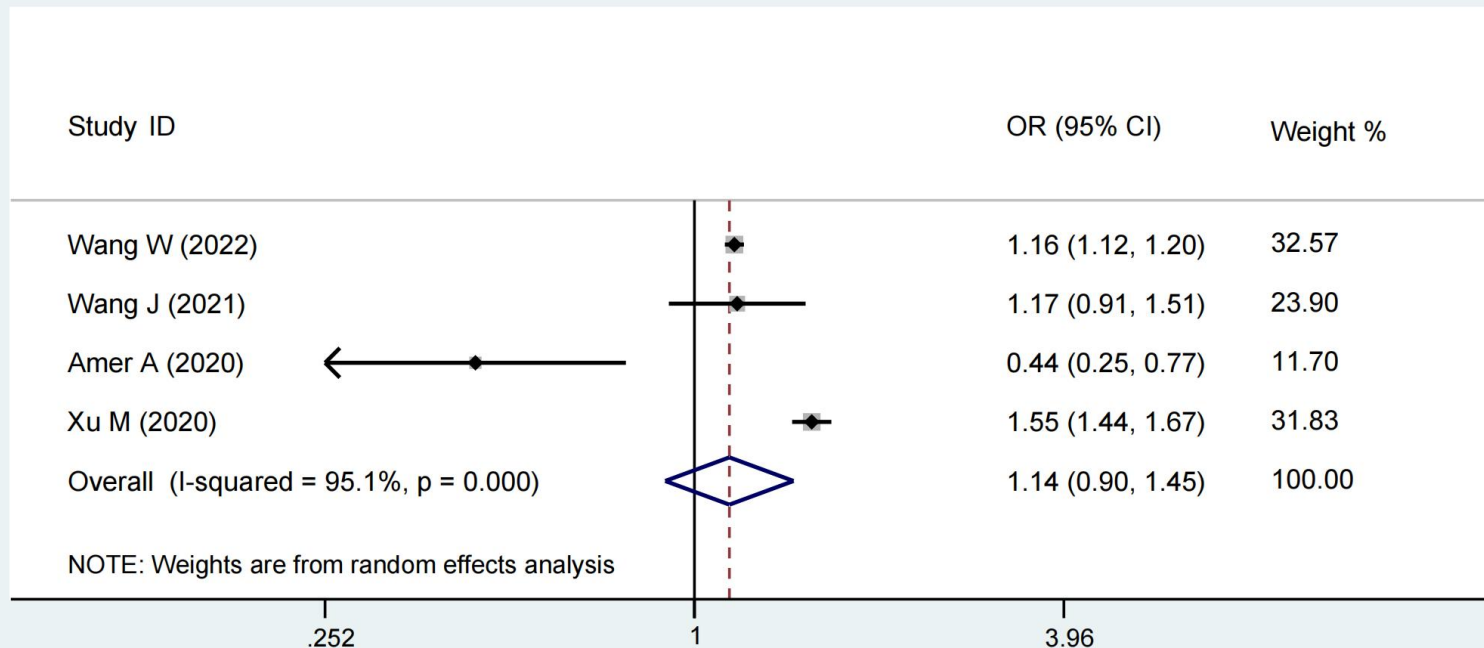

Supplement: Supplementary Materials — The paper includes supplementary tables 1–6 as supplementary materials. Their descriptions are as follows: Supplementary Table 1: quality of cohort and case-control studies. Supplementary Table 2: quality of cross-sectional studies. Supplementary Table 3: results of meta-regression analyses regarding the association of H. pylori infection with NAFLD in studies unadjusted for confounders. Supplementary Table 4: results of leave-one-out sensitivity analysis in studies unadjusted for confounders. Supplementary Table 5: results of meta-regression analyses regarding the association of H. pylori infection with NAFLD in studies adjusted for confounders. Supplementary Table 6: results of leave-one-out sensitivity analysis in studies adjusted for confounders. Supplementary Figure 1: forest plot of the proportion of H. pylori infection in patients with mild NAFLD. Supplementary Figure 2: forest plot of the proportion of H. pylori infection in patients with moderate NAFLD. Supplementary Figure 3: forest plot of the proportion of H. pylori infection in patients with severe NAFLD. Supplementary Figure 4: forest plots for unadjusted data from cohort studies. Supplementary Figure 5: forest plots for adjusted data from cohort studies. Supplementary Figure 6: H. pylori infection and the pathophysiological of MAFLD/NAFLD. [file 5521239.f1.zip › Supplementary Figure 1 (1).pdf]

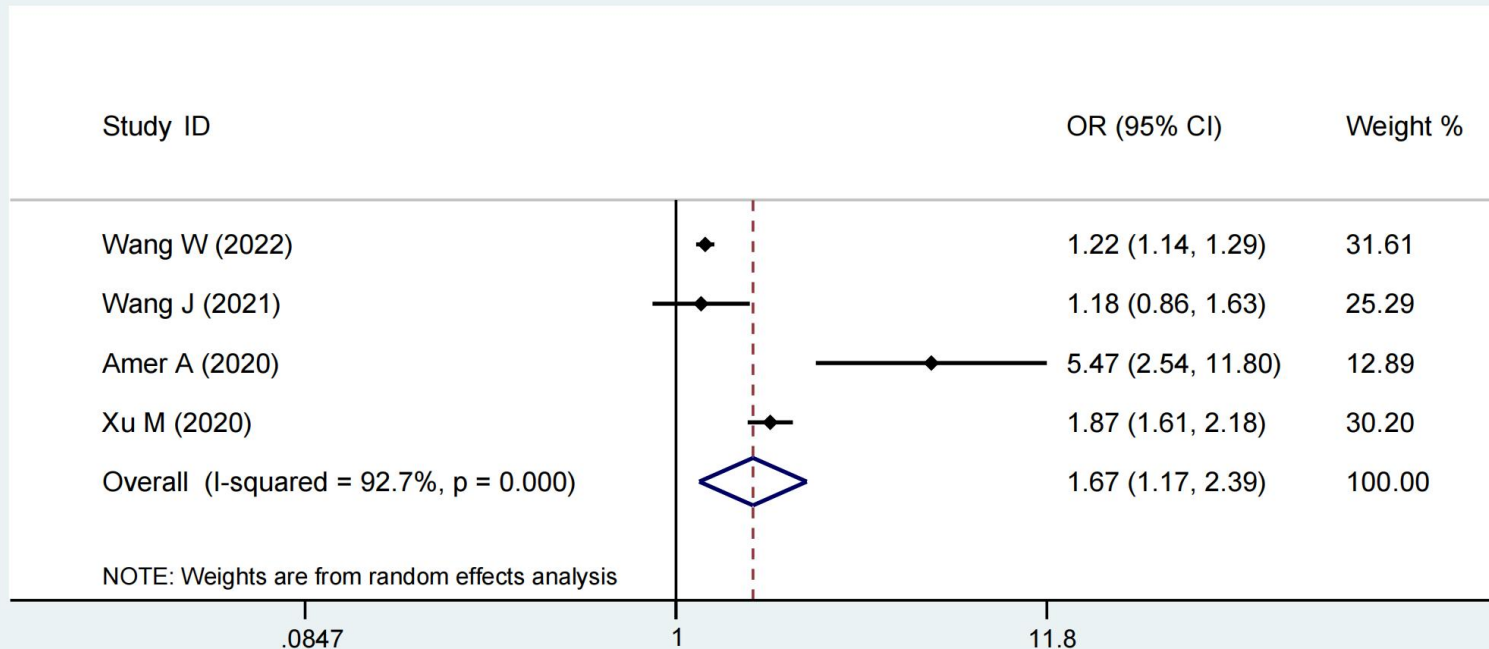

Supplement: Supplementary Materials — The paper includes supplementary tables 1–6 as supplementary materials. Their descriptions are as follows: Supplementary Table 1: quality of cohort and case-control studies. Supplementary Table 2: quality of cross-sectional studies. Supplementary Table 3: results of meta-regression analyses regarding the association of H. pylori infection with NAFLD in studies unadjusted for confounders. Supplementary Table 4: results of leave-one-out sensitivity analysis in studies unadjusted for confounders. Supplementary Table 5: results of meta-regression analyses regarding the association of H. pylori infection with NAFLD in studies adjusted for confounders. Supplementary Table 6: results of leave-one-out sensitivity analysis in studies adjusted for confounders. Supplementary Figure 1: forest plot of the proportion of H. pylori infection in patients with mild NAFLD. Supplementary Figure 2: forest plot of the proportion of H. pylori infection in patients with moderate NAFLD. Supplementary Figure 3: forest plot of the proportion of H. pylori infection in patients with severe NAFLD. Supplementary Figure 4: forest plots for unadjusted data from cohort studies. Supplementary Figure 5: forest plots for adjusted data from cohort studies. Supplementary Figure 6: H. pylori infection and the pathophysiological of MAFLD/NAFLD. [file 5521239.f1.zip › Supplementary Figure 2 (1).pdf]

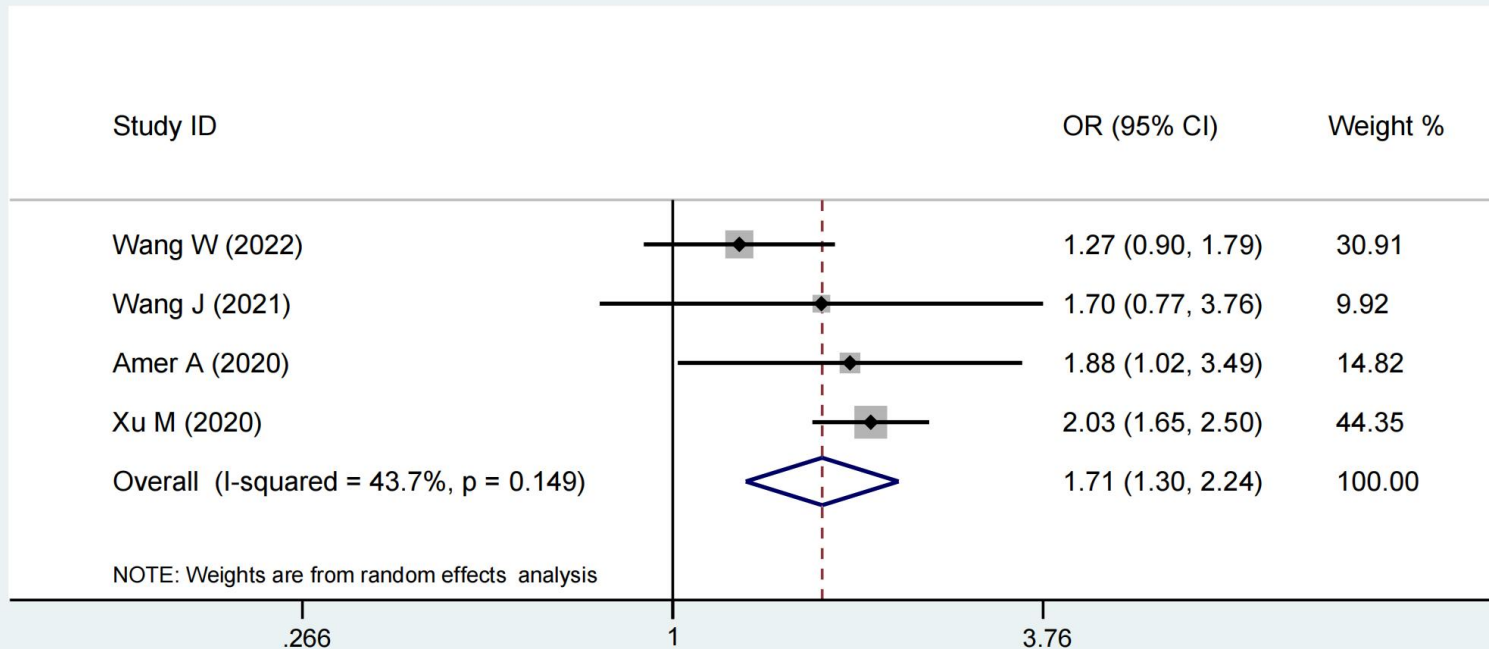

Supplement: Supplementary Materials — The paper includes supplementary tables 1–6 as supplementary materials. Their descriptions are as follows: Supplementary Table 1: quality of cohort and case-control studies. Supplementary Table 2: quality of cross-sectional studies. Supplementary Table 3: results of meta-regression analyses regarding the association of H. pylori infection with NAFLD in studies unadjusted for confounders. Supplementary Table 4: results of leave-one-out sensitivity analysis in studies unadjusted for confounders. Supplementary Table 5: results of meta-regression analyses regarding the association of H. pylori infection with NAFLD in studies adjusted for confounders. Supplementary Table 6: results of leave-one-out sensitivity analysis in studies adjusted for confounders. Supplementary Figure 1: forest plot of the proportion of H. pylori infection in patients with mild NAFLD. Supplementary Figure 2: forest plot of the proportion of H. pylori infection in patients with moderate NAFLD. Supplementary Figure 3: forest plot of the proportion of H. pylori infection in patients with severe NAFLD. Supplementary Figure 4: forest plots for unadjusted data from cohort studies. Supplementary Figure 5: forest plots for adjusted data from cohort studies. Supplementary Figure 6: H. pylori infection and the pathophysiological of MAFLD/NAFLD. [file 5521239.f1.zip › Supplementary Figure 3 (1).pdf]

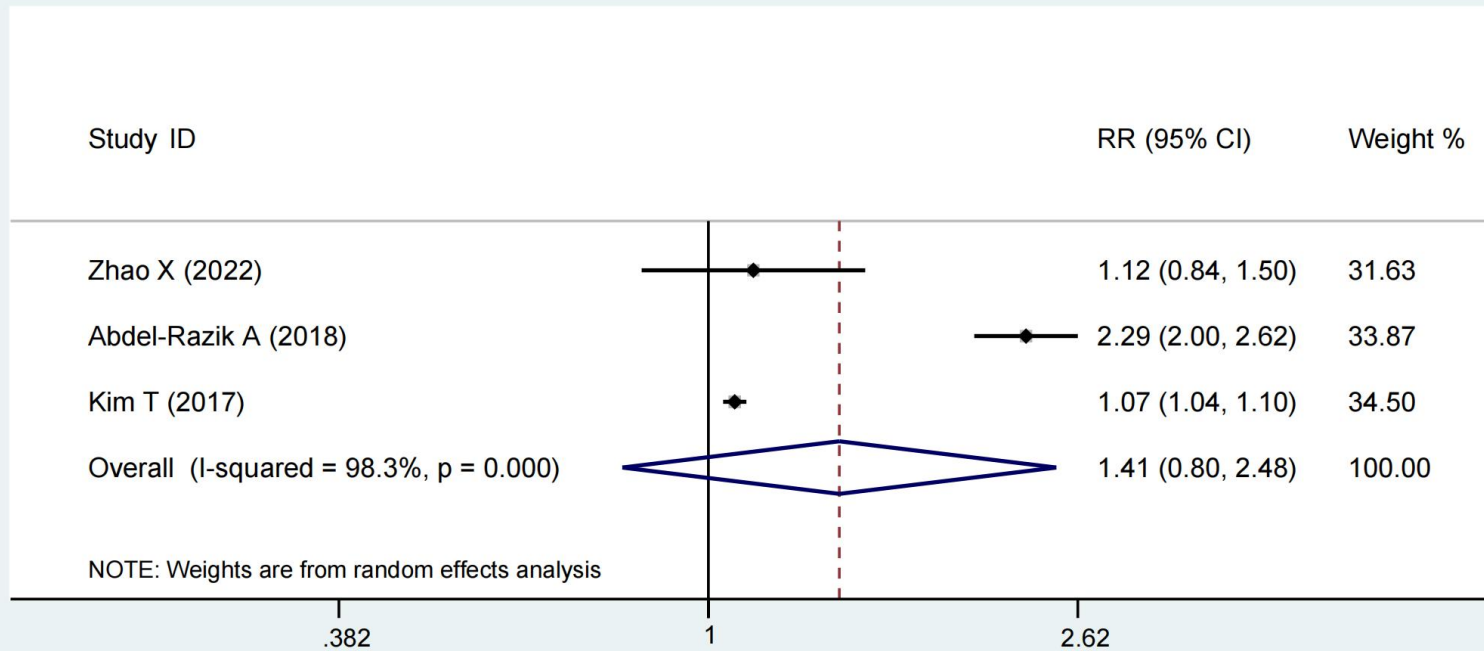

Supplement: Supplementary Materials — The paper includes supplementary tables 1–6 as supplementary materials. Their descriptions are as follows: Supplementary Table 1: quality of cohort and case-control studies. Supplementary Table 2: quality of cross-sectional studies. Supplementary Table 3: results of meta-regression analyses regarding the association of H. pylori infection with NAFLD in studies unadjusted for confounders. Supplementary Table 4: results of leave-one-out sensitivity analysis in studies unadjusted for confounders. Supplementary Table 5: results of meta-regression analyses regarding the association of H. pylori infection with NAFLD in studies adjusted for confounders. Supplementary Table 6: results of leave-one-out sensitivity analysis in studies adjusted for confounders. Supplementary Figure 1: forest plot of the proportion of H. pylori infection in patients with mild NAFLD. Supplementary Figure 2: forest plot of the proportion of H. pylori infection in patients with moderate NAFLD. Supplementary Figure 3: forest plot of the proportion of H. pylori infection in patients with severe NAFLD. Supplementary Figure 4: forest plots for unadjusted data from cohort studies. Supplementary Figure 5: forest plots for adjusted data from cohort studies. Supplementary Figure 6: H. pylori infection and the pathophysiological of MAFLD/NAFLD. [file 5521239.f1.zip › Supplementary Figure 4 (1).pdf]

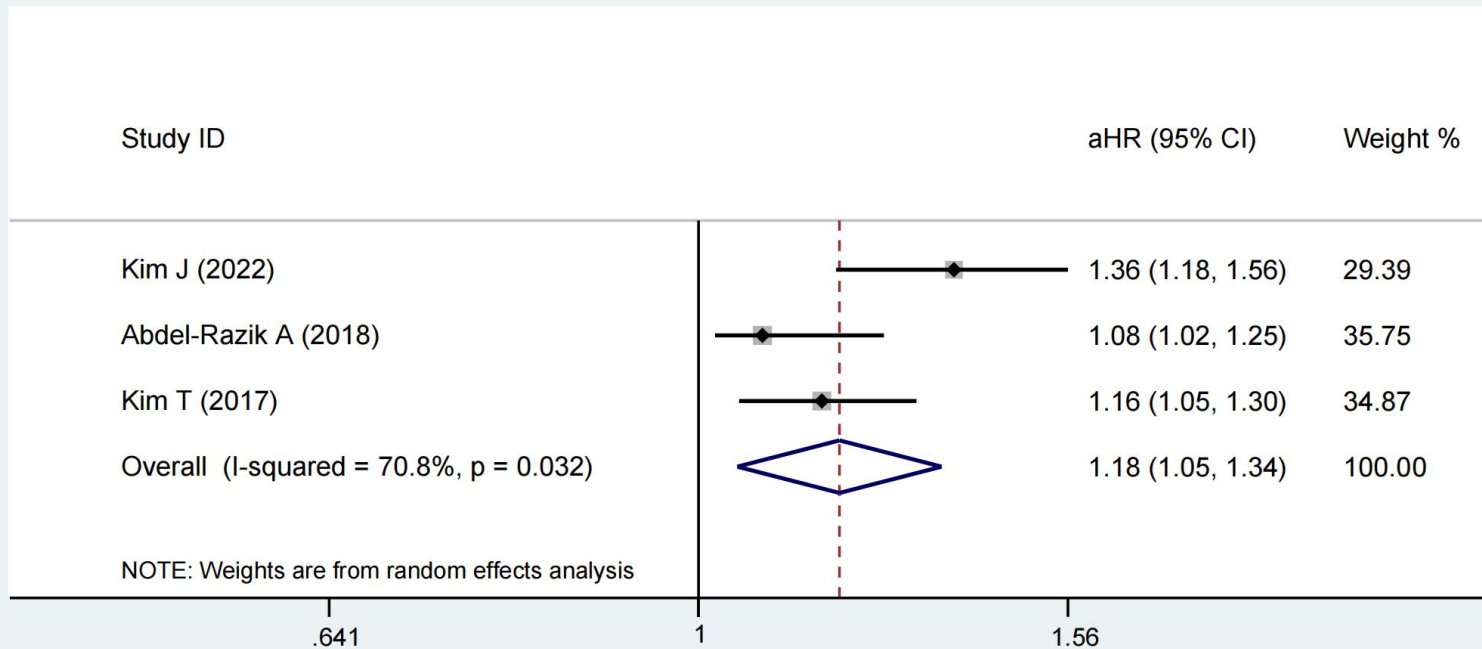

Supplement: Supplementary Materials — The paper includes supplementary tables 1–6 as supplementary materials. Their descriptions are as follows: Supplementary Table 1: quality of cohort and case-control studies. Supplementary Table 2: quality of cross-sectional studies. Supplementary Table 3: results of meta-regression analyses regarding the association of H. pylori infection with NAFLD in studies unadjusted for confounders. Supplementary Table 4: results of leave-one-out sensitivity analysis in studies unadjusted for confounders. Supplementary Table 5: results of meta-regression analyses regarding the association of H. pylori infection with NAFLD in studies adjusted for confounders. Supplementary Table 6: results of leave-one-out sensitivity analysis in studies adjusted for confounders. Supplementary Figure 1: forest plot of the proportion of H. pylori infection in patients with mild NAFLD. Supplementary Figure 2: forest plot of the proportion of H. pylori infection in patients with moderate NAFLD. Supplementary Figure 3: forest plot of the proportion of H. pylori infection in patients with severe NAFLD. Supplementary Figure 4: forest plots for unadjusted data from cohort studies. Supplementary Figure 5: forest plots for adjusted data from cohort studies. Supplementary Figure 6: H. pylori infection and the pathophysiological of MAFLD/NAFLD. [file 5521239.f1.zip › Supplementary Figure 5 (1).pdf]

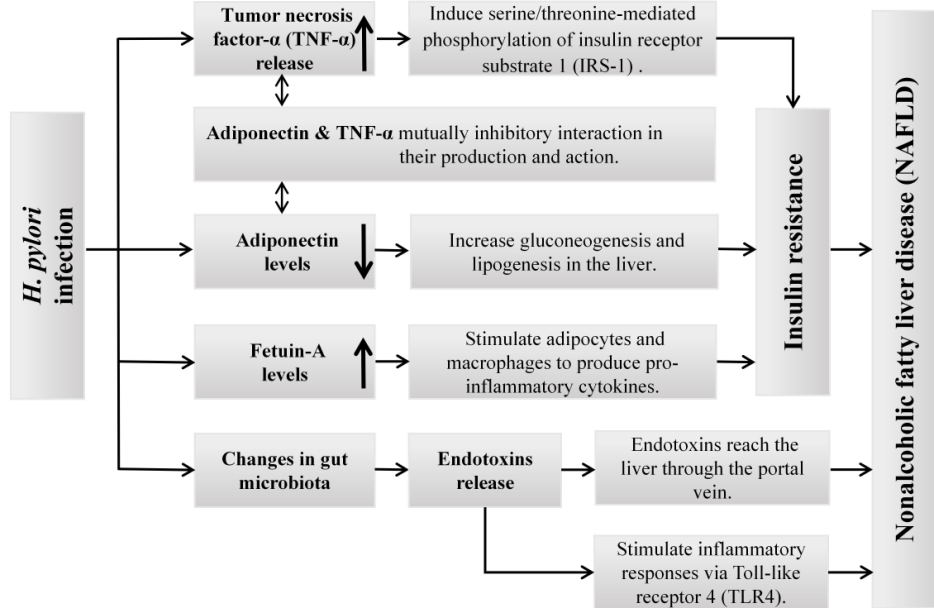

Supplement: Supplementary Materials — The paper includes supplementary tables 1–6 as supplementary materials. Their descriptions are as follows: Supplementary Table 1: quality of cohort and case-control studies. Supplementary Table 2: quality of cross-sectional studies. Supplementary Table 3: results of meta-regression analyses regarding the association of H. pylori infection with NAFLD in studies unadjusted for confounders. Supplementary Table 4: results of leave-one-out sensitivity analysis in studies unadjusted for confounders. Supplementary Table 5: results of meta-regression analyses regarding the association of H. pylori infection with NAFLD in studies adjusted for confounders. Supplementary Table 6: results of leave-one-out sensitivity analysis in studies adjusted for confounders. Supplementary Figure 1: forest plot of the proportion of H. pylori infection in patients with mild NAFLD. Supplementary Figure 2: forest plot of the proportion of H. pylori infection in patients with moderate NAFLD. Supplementary Figure 3: forest plot of the proportion of H. pylori infection in patients with severe NAFLD. Supplementary Figure 4: forest plots for unadjusted data from cohort studies. Supplementary Figure 5: forest plots for adjusted data from cohort studies. Supplementary Figure 6: H. pylori infection and the pathophysiological of MAFLD/NAFLD. [file 5521239.f1.zip › Supplementary Figure 6 (1).pdf]
